# Recent advancements to increase success in assisted reproductive technologies in cattle

**DOI:** 10.1590/1984-3143-AR2024-0031

**Published:** 2024-08-12

**Authors:** Marja Mikkola, Karolien Leen Jan Desmet, Elisabeth Kommisrud, Michael A. Riegler

**Affiliations:** 1 SpermVital AS, Hamar, Norway; 2 Geno SA, Hamar, Norway; 3 CRESCO, Centre for Embryology and Healthy Development, Department of Biotechnology, Inland Norway University of Applied Sciences, Hamar, Norway; 4 Holistic Systems Department, Simula Metropolitan Center for Digital Engineering, Oslo, Norway

**Keywords:** embryo technology, semen quality, fertility, breeding, machine learning

## Abstract

Assisted reproductive technologies (ART) are fundamental for cattle breeding and sustainable food production. Together with genomic selection, these technologies contribute to reducing the generation interval and accelerating genetic progress. In this paper, we discuss advancements in technologies used in the fertility evaluation of breeding animals, and the collection, processing, and preservation of the gametes. It is of utmost importance for the breeding industry to select dams and sires of the next generation as young as possible, as is the efficient and timely collection of gametes. There is a need for reliable and easily applicable methods to evaluate sexual maturity and fertility. Although gametes processing and preservation have been improved in recent decades, challenges are still encountered. The targeted use of sexed semen and beef semen has obliterated the production of surplus replacement heifers and bull calves from dairy breeds, markedly improving animal welfare and ethical considerations in production practices. Parallel with new technologies, many well-established technologies remain relevant, although with evolving applications. *In vitro* production (IVP) has become the predominant method of embryo production. Although fundamental improvements in IVP procedures have been established, the quality of IVP embryos remains inferior to their *in vivo* counterparts. Improvements to facilitate oocyte maturation and development of new culture systems, e.g. microfluidics, are presented in this paper. New non-invasive and objective tools are needed to select embryos for transfer. Cryopreservation of semen and embryos plays a pivotal role in the distribution of genetics, and we discuss the challenges and opportunities in this field. Finally, machine learning (ML) is gaining ground in agriculture and ART. This paper delves into the utilization of emerging technologies in ART, along with the current status, key challenges, and future prospects of ML in both research and practical applications within ART.

## Introduction

Sustainable production of enough nutritious food for the growing world population poses a significant challenge to society and the importance of national self-sufficiency has been underscored by the COVID-19 pandemic in numerous countries. Animal breeding and genetic diversity form the foundation of the global food supply and for food production regionally and locally. In this context, efficient reproduction is crucial for sustainability in dairy and beef production.

Traditionally, quantitative breeding methods with progeny testing of large offspring groups for desirable phenotypes have been applied for selection of animals possessing economically important traits. The introduction of genomic selection (GS), based on next generation sequencing using panels of increasing numbers of single nucleotide polymorphisms (SNPs) distributed throughout the genome (Meuwissen et al., 2016), has changed breeding from a patient procedure to the pursuit of genetic progress in the shortest possible time. This recent shift in the industry towards the intensive use of young GS bulls has increased the urgency of predicting male fertility, as there is no time to obtain field fertility results before the next generation of GS bulls is introduced in semen production (Fair and Lonergan, 2018), and the search for biomarkers for the onset of puberty and sexual maturity is urgent (Bremer et al., 2023a).

Genomic selection, coupled with established and emerging assisted reproductive technologies (ART), has facilitated the inclusion of females in breeding programs. These technologies are essential for reducing generation intervals further. Multiple ovulation and embryo transfer (MOET), ovum pick-up (OPU) and *in vitro* production of embryos (IVP), combined with cryopreservation are well established technologies. However, pregnancy rates of IVP embryos are still below their *in vivo* derived counterparts, ranging between 45-50%. Many IVP embryos may look morphologically normal, but have fewer blastomere numbers, have a reduced hatching and implantation potential (Wydooghe et al., 2014), and may contain chromosomal aberrations (Tšuiko et al., 2017) that lead to embryonic losses. These deviations are caused by the suboptimal conditions of the *in vitro* environment, in the absence of the maternal genital tract. Epigenetic changes occur more frequently in IVP embryos, leading to Large/Abnormal Offspring Syndrome (LOS/AOS) (for review, see Nava-Trujillo and Rivera, 2023). As ARTs play a significant role in enhancing genetic gain, the collaboration of industry and academia resources is imperative for further increase of the sustainability of dairy and beef production. Despite the pivotal role that ART can play, the full potential of these technologies is currently not fully realized due to limitations imposed by social perceptions and regulatory policies. The implementation of precision breeding, incorporating gene editing, holds the promise of transforming the breeding industry. As technology is already in existence, but its application on food production animals is frequently restricted, enormous opportunity costs are running due to the failure to take advantage of the potential of gene editing in the production of disease-resistant and economically efficient livestock (van Eenennaam et al., 2021). In this paper, we discuss a number of technologies currently in commercial use or demonstrating promising potential for application in the field. More advanced technologies such as genome editing and cloning, which have yet to receive global acceptance, will be beyond the scope of this paper.

In the first part of this article, we will discuss current approaches and trends in cattle breeding with a focus on reducing the generation interval and accelerating genetic progress through ART. Central to this process is the selection of animals with the highest genetic merit based on genomic analyses. Beyond genetic criteria, AI bulls and embryo donors must meet various physiological criteria to successfully advance the next stages of the breeding process. The overarching goal is to ensure that the germ cells from these elite animals produce the next generation as quickly and efficiently as possible. This process consists of i) prediction and possible manipulation of sexual precocity, ii) selection of the most competent gametes, iii) optimal processing methods for the gametes and embryos, and finally, iv) managing fertility of the recipients of semen or embryos. Artificial intelligence, already making strides in various industries, including agriculture and ART, is already applied in many of the technologies discussed here (Hanassab et al., 2024; Riegler et al., 2021). Therefore, in the second part of the paper, we delve into the current status and future prospects of artificial intelligence in ART.

## Shortening the generation interval

### Selecting young bulls

Good male fertility is essential to succeed economically in agriculture, thus a prerequisite for sustainable food production. Breeding companies currently select bulls based on breeding soundness evaluation (BSE) focusing mainly on examination of a few characteristics of spermatozoa such as motility and morphology. It has been shown that bulls having passed BSE criteria still turn out to show huge differences in conception rates, prompting the need for multiple parameters to predict fertility (Kumaresan et al., 2017). Measurement of scrotal circumference (SC) is a widely used selection criterion for bulls in breeding programs, and larger SC has been associated with earlier onset of puberty and increased sperm production (Kastelic, 2014; Waite et al., 2019). BSE systems vary considerably and have different applications to dairy and beef bulls. Common parameters assessed by BSE are SC, libido, and sperm quality parameters (Barth, 2018). In some countries, the BSE systems are implemented on the national level, whereas in other countries, implementation depends entirely on breeding companies and their routines. Recently, a small survey on selection criteria for AI bulls was conducted in some European and North American breeding companies that applied genomic selection (Bremer, 2023). The survey revealed differences in traits and parameters applied in their BSE. There were, e.g., differences in semen quality thresholds as well as for the practice of applying SC measurements. Most, but not all, companies used SC as a routine screening for bulls before entering semen production. Machine learning (ML) is a novel approach for, e.g., predicting bull sperm quality (Hürland et al., 2023). More specifically, in the area of ART for cattle, the study by Bremer et al. (2023b) demonstrated the potential to automate SC measurements in bulls using 3D imaging and convolutional neural networks (CNN). In this study the SC of Norwegian Red bulls were measured in two ways. First, SC was measured manually by applying scrotal tape at four–time points for individual bulls: 3-5 months upon arrival and later at approximately 6, 9 and 12 months of age. Thereafter, each bull’s scrotum was photographed using a handheld device consisting of an Intel Real Sense d415 camera connected with a tablet by a stick (Afridi et al., 2022). The camera was carefully placed on the floor between the bull’s legs. The proposed method offers the advantages of non-contact measurement, improving safety for veterinarians and technicians and reducing stress on the animals, as well as increasing efficiency of BSE. However, the accuracy of this method depends on the quality of the 3D images, especially sufficient lighting conditions. Additionally, the model performs optimally on bulls older than 6 months due to their more pronounced scrotal development. Key findings of this study indicate that the CNN-based method can achieve SC measurements comparable to manual methods.

### Selecting oocyte/embryo donors

Variation in embryo production between donors is a serious challenge for cattle breeders. The outcome of ART is highly affected by the individual physiological characteristics of the animal, such as the variability of ovarian antral follicle population. Antral follicle count (AFC) is used to predict the ovarian response to gonadotropin-based treatments. Once superovulation is initiated, the number of small antral follicles is highly correlated to the number of transferable embryos (Ireland et al., 2007). Anti-Müllerian hormone (AMH), also referred to as Müllerian inhibiting substance (MIS), is produced by granulosa cells of small antral follicles and is considered as a an endocrine biomarker of ovarian follicular reserve (Batista et al., 2014; Ireland et al., 2011) and response to superovulation treatments (Monniaux et al., 2010; Souza et al., 2015). Scientific reports have shown a high correlation between AMH levels and number of large follicles after superstimulation, number of CL after superovulation and number of embryos collected using MOET protocols in both dairy and beef cows (Hirayama et al., 2012); Rico et al., 2012). Therefore, measuring AMH before enrolling females in superovulation programs would likely allow practitioners to increase the number of embryos produced and thereby reduce the costs per embryo produced. Similarly, AMH can be an interesting endocrine marker to select donors with the greatest potential for OPU-IVP. Plasma AMH in *Bos indicus* and *Bos taurus* heifers is positively correlated with total follicles aspirated, total cumulus oocytes complexes (COCs) retrieved, number of COCs cultured, and number of embryos produced per OPU session. However, plasma AMH concentration did not alter the ability of COCs to reach the blastocyst stage (Guerreiro et al., 2014).

Identification of heifers with a high potential for embryo production at birth or weaning would also be advantageous for breeders. An experiment in Maine-Anjou beef heifers showed that the level of AMH increased from one to three months of age to six months of age and then slowly decreased from seven to 12 months of age. The onset of puberty was one year for this breed. Similar changes were observed in Holstein heifers, in which the level of AMH was increased until two months of age, started to decline in the fifth month, and then stabilized when the puberty started at the age of eight to nine months (Monniaux et al., 2012). This suggests that the level of AMH is high during early life in cattle, but there is variation due to the age of puberty onset in different breeds. Several studies have illustrated that IVP performance, including antral follicle population, the number of recovered oocytes, and the number of embryos produced *in vitro*, can be predicted by AMH in very young calves, ranging from the age of two to seven months (Batista et al., 2014; Krause et al., 2022). The determination of plasma AMH may facilitate the selection of very young animals in breeding programs, overcoming the technical difficulty of ultrasound examination of the ovaries of potential young donors. This could again, in combination with genomic selection of young animals, allow faster genetic gain by markedly decreasing generation intervals.

Although the use of AMH as a biomarker for potential embryo donors seems promising, several challenges have been identified that limit its applicability. Despite the fact that AMH concentration exhibits high repeatability for an individual, it has a high variability between individuals (Ribeiro et al., 2014; Souza et al., 2015). Due to this wide variation in AMH levels from herd to herd, no benchmark AMH level has been established yet. Instead, animals are compared with their contemporary groups to categorize them as having high or low AMH concentrations. Furthermore, breed impacts AMH levels with *Bos indicus* having the highest AMH levels, followed by beef breeds and *Bos taurus* dairy breeds (Jersey, Jersey-Holstein crossbreeds and Holsteins) (Batista et al., 2014; Ribeiro et al., 2014). Several maternal factors during gestation are found to influence AMH concentrations in offspring. Excessive growth prior to conception and during early gestation of nulliparous heifers has been shown to reduce AMH concentration in their offspring (Thomson et al., 2024). Similarly, undernutrition in the first trimester can cause a reduction in antral follicle count and plasma AMH even when nutritional restriction does not affect gross development of the offspring (Mossa et al., 2013). Maternal milk production and chronically high somatic cell count may produce daughters with smaller ovarian reserves, lower plasma AMH and suboptimal fertility as adults (Ireland et al., 2011; Mobedi et al., 2024). Another study showed that heifers born to mothers exposed to heat stress in early gestation had smaller ovarian reserves and lower AMH concentrations, but no effects on fertility were identified at first conception (Succu et al., 2020). These examples show that the management of dams to potential embryo donors may influence their reproductive performance and could even improve the performance by feeding strategies.

### Embryo production and generation interval

#### Embryo production from young donors

In the pursuit for a shorter generation interval and accelerated genetic gain, the age of the gamete donors, both females and males, is critical. On the female side, embryos should be produced from as young donors as possible. However, successful *in vivo* embryo production requires a sufficient precocity state of the donor to be feasible. Instead, IVP from oocytes recovered from prepubertal heifer or juvenile calf oocytes has been well established and offers an approach in the battle against time (for a comprehensive review, see Baldassarre, 2021). Oocytes can be collected from as young as 2-month-old calves with a laparoscopic ovum pick-up approach (LOPU). Where the quantity of recovered oocytes from 2- to 6-month-old heifer calves is generally higher than those of pubertal heifers, the developmental competence of calf oocytes is lower than for mature animals, resulting in lower blastocyst rates. Hormonal priming of ovaries with external gonadotrophins, either FSH alone or FSH in combination with eCG, is necessary to increase the follicular size and oocyte developmental competence. Extending the hormonal priming of ovaries from the standard approximately 48 hours to 72 hours increases follicular diameter, enhances cytoplasmic maturation and thus improves the developmental rate of oocytes after LOPU (Baldassarre, 2021; Currin et al., 2017; Kauffold et al., 2005). Modifications are needed in the protocols for hormonal stimulation as well as laboratory procedures to decrease the rate of polyspermia (Baldassarre, 2021) and increase the production of blastocysts from juvenile oocytes.

Despite the fact that laparoscopic OPU can reduce the generation interval by up to more than half a year compared to transvaginal OPU, the vast majority of genetics companies in Europe are currently not routinely implementing LOPU in their breeding programs. Ethical concerns and consumer acceptance are among the reasons for abstaining from LOPU. Instead, most European companies currently consider adequate body size for the transvaginal approach, and some companies even consider the onset of estrous cyclicity, as criteria for *in vitro* embryo production. In some European countries, such as Finland, oocyte collection from donors younger than 6 months is currently prohibited by animal welfare regulations. In turn, in many regions producing large numbers of cattle embryos, such as North America and Brazil, the collection of oocytes with the laparoscopic approach is actively used to enhance the rate of production of progeny from selected elite females. However, currently there are no global statistics available that differentiate between laparoscopic and transvaginal OPU.

#### Embryonic stem cells

*In vitro* breeding (IVB) is another new strategy that will notably accelerate genetic improvement in livestock populations. Although generation intervals have decreased significantly after the implementation of genomic selection, several factors, such as gestation length and arrival to sexual maturity, maintain these intervals constrained to years (García-Ruiz et al., 2016). IVB can significantly reduce the generation interval by combining GS with embryonic stem cell (ESC) derivation and *in vitro* differentiation of germ cells from pluripotent stem cells. Recent advances in the derivation of ESCs from bovine blastocysts (Bogliotti et al., 2018) and the *in vitro* generation of germ cells from ESCs in mice (Hikabe et al., 2016) have paved the way to accelerate the genetic improvement of livestock populations even more. This method would start with an estimate of genotypic values associated with productive traits of interest similar to GS. Embryos would then be generated *in vitro* from males and females with high genetic merit in the population. ESC cultures would be produced from the inner cell mass (ICM) of the blastocysts, subsequently genotyped and estimated embryonic breeding values would be calculated. Tens or hundreds of cell lines with high genetic merit could be selected from the candidates and would be used for the generation of functional gametes. These gametes could then be included in a new round of IVP, ESC selection, and germ cell differentiation. The main advantage of this strategy lies in the time it takes to carry out each round of IVB. Since an IVP round followed by ESC derivation takes approximately 4 weeks in cattle, and germ cell differentiation takes about 2 or 3 months in mice, a breeding round through IVB could be completed in around 3 to 4 months. Implementation of this new technique would allow substantial improvements in production efficiency in a significantly shorter time, resulting in fewer animals needed to provide larger amounts of animal products, and thus decrease the footprint of livestock on the environment. However, more studies are required to optimize its practical application in breeding programs. It may be tempting to obtain the maximum genetic potential in just a few embryos and to use these embryos to breed future generations. This could result in significant losses in genetic variance within populations and should be closely monitored. Another concern includes epigenetic changes that may result from continuous cell and embryo culture. *In vitro* embryo production in bovine species has been associated with detrimental fetoplacental development, such as lower pregnancy rates, early embryonic loss, prolonged gestation, and fetal overgrowth (Duranthon and Chavatte-Palmer, 2018; Nava-Trujillo and Rivera, 2023). Long-term monitoring of offspring derived from IVB would also be necessary before implementing this technique in farm animal breeding programs.

## Making and selecting fertile semen doses

### Predicting the fertility of semen

Although several studies have shown associations between sperm quality parameters and field fertility, there is still no single *in vitro* analysis that reliably and repeatedly can predict the true fertilization potential of an ejaculate or a semen sample. Traditional *in vitro* evaluation of sperm quality includes e.g. sperm motility and velocity, assumed to be important for fertilization capacity. For example, several studies on motility parameters have shown both to be associated with field fertility and not. Motility parameters are characterized as compensable sperm traits and the varying number of sperm cells in an AI dose can be a bias when comparing studies, which could also apply for other compensable sperm traits.

Computer assisted sperm analysis (CASA) has been demonstrated to be important for objective assessment of motility and motility patterns in mammals (Amann and Waberski, 2014). However, specific sperm subpopulations, cluster analyzes, are more likely to be important in predicting fertility (Leemans et al., 2019). Important is that the motility results obtained from a CASA system depend on the specific system applied and the settings. As an ejaculate consists of distinct sperm populations, an approach to selecting the desired ones is to use different centrifugation methods, for example single layer centrifugation (SLC) with species-specific colloid formulations (Morrell et al., 2009). Several studies have shown an improved effect on the quality *in vitro* of semen samples (Nongbua et al., 2017), however, *in vivo* results have not been demonstrated and there is a question of how many possible fertile sperm are lost during the procedure. The technique has potential to be applied to improve, for example, sperm cell sex sorting and has recently been demonstrated as a possible method for bacterial reduction in bull semen without the use of antibiotics (Cojkic et al., 2024).

In recent decades, omics such as high-throughput transcriptomic, proteomic, and metabolomic analysis of seminal plasma and sperm cells are applied broadly in science (Talluri et al., 2022). Recently, it was demonstrated that the combination of DNA fragmentation index (DFI) and intracellular sperm concentrations of selected metabolites and elements (aspartic acid, Fe and Zn) in the viable population of frozen-thawed semen were predictive of bulls’ fertility measured as 56-day non-return rates (NR56) (Narud et al., 2020). Further, differential methylation analysis showed that spermatozoa from bulls of low NR56 were hypermethylated in comparison to bulls of high NR56. Pathway analysis revealed that genes annotated with differentially methylated cytosines could be of importance in biological pathways related to bull fertility (Narud et al., 2021). Traditionally, spermatozoa was believed to be transcriptionally silent with the only purpose of transporting and delivering the paternal genome to the oocyte (Grunewald et al., 2005). However, an increasing number of studies have shown that mature mammalian sperm carry thousands of RNAs. The roles of microRNAs and small interfering RNAs in mammalian spermatogenesis in relation to argonaute proteins were reviewed by Hilz et al. (2016). Later, Sellem et al. (2020) provided a comprehensive overview of bull sperm small non-coding RNAs, which were found to be differentially expressed across breeds. Several studies have paved the way for future work on molecular biology and omics; however, the ultimate biomarker(s) for bull fertility is still to be explored further.

A commercially applied approach to identify high fertility semen batches has been recently presented. This approach to predict the fertility of a semen dose is based on medium infrared (MIR) spectroscopy of pre-diluted semen and frozen thawed semen at the batch level (Ghazouani et al., 2020). Briefly, after thawing and washing of the spermatozoa, the metabolic profile of the semen sample is analyzed on the basis of its spectrum using Fiber Evanescent Wave Spectroscopy (FEWS). Therefore, the diagnosis of fertility level is based on the analysis of the molecular characteristics of the sample, validated in a reference population of 41,000 inseminations for frozen application, rather than predetermined markers (Guillaume et al., 2017). The fertility of semen doses validated by this patented technology is applied for the production of ultra-fertile semen doses registered under the trademark Fertimax©, showing on average eight percentage points higher non-return rate at 90 days after AI (Mastergen, 2024).

## Semen processing techniques

### Semen processing to improve quality and extend the lifespan of spermatozoa

Prolonging the shelf life of sperm cells is assumed to make the timing of AI less critical and is a possible approach to increasing fertility. It was first demonstrated that encapsulation of bovine sperm in alginate capsules is compatible with healthy sperm survival during storage of encapsulated sperm at 37 °C (Nebel et al., 1996). Later, it was demonstrated that encapsulated spermatozoa retain fertilizing capacity after AI (Munkittrick et al., 1992; Nebel et al., 1993; Vishwanath et al., 1997), also when AIs were performed in proestrus (Nebel et al., 1996). The patented SpermVital technology (Kommisrud et al., 2012), uses a fundamentally different approach to immobilize spermatozoa than the previous described methods as spermatozoa are immobilized within a solid gel network made of calcium alginate gel in combination with cryopreservation. Spermatozoa are immobilised in the alginate gel to enable their gradual release following insemination. It has been shown that inseminations performed with SpermVital semen at normal timing relative to natural occurring estrous signs show comparable fertility with conventionally produced semen (Standerholen et al., 2015; Berg et al., 2018). Furthermore, AI performed early in synchronized estrus with a single SpermVital semen dose resulted in equal conception rates as double AI with conventionally processed semen inseminated on two consecutive days (Alm-Kristiansen et al., 2017). With uterine endoscopy, visualization of gel remnants in the uterus 3, 6, 20 and 24 hours after AI, demonstrates the potential release of spermatozoa during this period (Berg et al., 2020), and the possible advantages of combining this immobilization technique, not only with cryopreservation, but also with other semen processing technologies.

### Sexed semen

Several authors have recently comprehensively reviewed the technology and commercial application of sexed semen (Garner and Seidel, 2008; Seidel, 2014; Vishwanath and Moreno, 2018). Historically, a number of various approaches have been presented to semen sorting, based on the difference in the kinetics and physical properties of the sperm or immunology. Approaches such as the Percoll gradient (Kaneko et al., 1983), swim-up (Han et al., 1993) and albumin gradient (Ericsson et al., 1973) have been studied; however, none of them has reached the accuracy and efficiency required for large-scale commercial use. So far, the only commercially feasible approach is based in the difference of the DNA-content of X and Y-chromosome bearing spermatozoa, quantified by differential fluorescence of Hoechst 33342 stained cells and sorted by flow cytometry. Currently, there are two commercial approaches exploiting the DNA content difference of X and Y-spermatozoa. Following quantification of the fluorescence, the cells are either sorted in X and Y-fractions, or the spermatozoa of undesired sex are ablated (Faust et al., 2016).

The challenges related to the technology lie in the relatively small number of cells per dose, restricted by the economical feasibility. Physical and mechanical stress posed to cells during the sorting procedure, as well as the low number of cells in straw contribute to compromised pregnancy rates compared to conventional semen. Since the early years of commercialization, the efficiency of the technology has increased markedly, both in terms of production efficiency and pregnancy outcomes. A recent comprehensive meta-analysis (Reese et al., 2021) demonstrates a reduction of 23% in pregnancy rates compared to conventional semen. When increasing the dose to 4 million sperm, the difference diminished to 16% .

Today, millions of doses of sexed semen are produced annually from thousands of sires worldwide (Hasler, 2023). In many countries, a rapid increase in the sales of sexed semen can be observed in the past 6-7 years. In UK, the turning point was in 2020, when sexed semen sales exceeded conventional semen in dairy breeds. In 2023, the proportion of sexed dairy semen represented already 76.5% of all dairy semen sales Agriculture and Horticulture Development Board (AHDB, 2023). Increased use of sexed semen has several positive consequences on the cattle industry. In dairy herds, when genetically superior females of the herd are inseminated with sexed semen for replacement heifer production, consequently, average to low-ranked females can be inseminated with beef semen. This has marked consequences on dairy producers’ economy as well as animal welfare. The welfare consequences of using sexed semen are both direct and indirect. The direct consequences include the reduced dystocia and stillbirth by use of X-sorted semen (Norman et al., 2010). Indirect consequences are due to the increased use of beef semen, driven by sexing of semen in dairy, thus helping to overcome the production of surplus male dairy calves and improving the welfare of the calf (Crowe et al., 2021).

Semen sexing technology has had and will continue to influence the cattle industry by accelerating genetic gain and increasing sustainability. The current rapid development in technologies such as proteomics and nanotechnology can potentially provide alternative tools in the toolbox of sex sorting in the future by utilizing the differences in membrane proteins of X and Y sperm (Quelhas et al., 2021).

### Heterospermic insemination

The use of beef semen on dairy is rapidly increasing worldwide (Berry, 2021). Recently, heterospermic or pooled semen on beef breeds has been implemented on many markets. However, detailed statistics on this specific semen product are difficult to access, partly due to a delay in technical solutions for the registration of semen doses from several donors. In European Union, specific regulations on movements of pooled semen were laid in 2021. The interest in pooled semen lies in its potentially higher fertility. When mixing ejaculates from typically two to three individuals, the final product will contain sub-populations of sperm with varying kinematics and metabolism. Thus, it is assumed that heterospermic insemination provides a wider insemination window than homospermic insemination.

However, until now the number of large field trials comparing pregnancy outcomes after insemination with homospermic and heterospermic semen is scarce and more studies are needed. Most studies show no effect; some show slightly beneficial effects on fertility when using heterospermic semen (Diskin, 2018). It seems likely that rather than increasing the overall fertility of the pooled semen dose above the average of individuals in the mix, the beneficial effects result from a possibly low fertility bull being “back-upped” by the higher ones. In addition to evaluating pregnancy outcomes in insemination trials, *in vitro* studies may reveal new insights. Recently, findings from a study on sperm bioenergetics suggested an unexplained compensatory mechanism after co-incubation of heterospermic semen for 24 hours in room temperature (Agostini Losano et al., 2023). The basal respiration of the pooled semen of two individuals was higher but the mitochondrial membrane potential was lower than for these bulls individually. On the other hand, a different combination of two bulls was metabolically identical to its individuals, suggesting that different combinations may behave differently over time.

### Liquid semen

Since the invention of the cryoprotective properties of glycerol in 1949 (Polge et al., 1949) and subsequent development of cryopreservation protocols for bull semen, frozen semen has become the industry standard. It is estimated that 95% of the global semen market is based on frozen semen (Thibier and Wagner, 2002; Wiebke et al., 2022). However, there are a few countries that deviate from the mainstream, such as New Zealand and Ireland, that use liquid semen during their highly concentrated breeding season.

However, despite that liquid semen in the global perspective represents only a hardly notable proportion of the volume of the semen market, there is evidence of growing interest toward it among genetics companies. Liquid semen can respond to the challenge of producing abundant doses from young bulls whose quantity and quality of semen do not meet the quality criteria after freezing. Furthermore, overcoming the 30 days post-collection holding period, mandatory for frozen bovine semen in the European Union, makes liquid semen an attractive aid in shortening the generation interval in European breeding companies. This approach is implemented in many embryo programs, facilitating the early use of the young bulls’ first ejaculates when used for IVF and embryo production on high genomic heifers.

In addition to shortening the generation interval, another benefit of liquid semen is related to the avoidance of cryoinjuries, thus allowing a reduction in the number of sperm per dose. Typically, 2 to 10 million spermatozoa are used for liquid semen doses, thus allowing the production of more doses per ejaculate compared to frozen semen, for a review see Wiebke et al. (2022). It is possible to achieve equal or greater conception rates with more diluted semen, resulting in a higher calf output per ejaculate compared to frozen semen. The longer survival time in the female genital tract is demonstrated in studies, where improved pregnancy outcomes of liquid semen are more apparent when the time interval between AI and ovulation is prolonged (Borchardt et al., 2018; Tippenhauer et al., 2021; Wiebke et al., 2023).

The downsides of liquid semen are its relatively short storage time and the need for a specific distribution system. One could also underline the possible increased risk of disease transmission via semen. The health status of the donors has not been monitored for 30 days after semen collection as in the case of frozen semen in the EU. Testing of bulls and straws for infectious diseases is challenging in such short notice that is needed in case of liquid semen. Currently, most actors are using liquid semen for 3 to 4 days after production, until fertility starts to decline below the level of frozen semen. To increase the lifespan of semen, several commercial extenders have been developed. Compared to frozen semen, liquid semen is more subject to microbiological challenges during storage time, as well as oxidative stress for spermatozoa. After several days of storage, the sedimentation of cells in the straw is inevitable, unless a gel-like extender is used. In our studies, after one day of storage, nearly the total number of cells is condensed in the lower half of the straws when a liquid extender is used, whereas processed with SpermVital extender, half of the cells can be found in each half of the straw, thus demonstrating the extender properties to prevent sedimentation (unpublished data).

## Making and selecting the best embryo

### Ovarian stimulation

In 2022, a total of 69,240 embryo collections were reported worldwide, a superovulation treatment preceding each of these procedures (Viana, 2023). Furthermore, ovarian stimulation preceded 41% of the 344,240 OPU sessions reported. In Europe and North America, almost 90 and 70% of donors, respectively, are stimulated prior to OPU. Based on this information, it can be concluded that a minimum of 140,105 FSH treatments were administered to oocyte donors. Considering that not all countries report their commercial ET-activities, and that among countries reporting, not all do stratify their data so that it allows detailed information on stimulated OPU sessions, the total number of ovarian stimulations is presumably far higher than 210,000 and is likely to continue growing (Viana, 2023).

During the past decades, the vast majority of ovarian stimulations have been performed using porcine pituitary FSH (pFSH) and the commercial selection as well as availability of pFSH has been relatively stable. However, since the COVID-19 pandemic, regional shortages of pFSH products have plagued breeding companies and ET-practitioners. In many markets, the cost of FSH has nearly doubled in the past few years. This situation, as well as the need for frequent twice-daily administration of pFSH has increased the interest towards alternative products for superovulation, namely recombinant FSH (rFSH) and eCG. Despite that the early reports of superovulation with rFSH were published in late 1980’s (Looney et al., 1988), following years and decades did not show marked progress in broad commercial application of rFSH. However, recently the frequency of studies has been increasing, reviewed comprehensively by Baruselli et al. (2023).

The benefits of rFSH include its purity and consistent bioactivity; increased biosecurity, as the final product can be free of animal derived proteins; as well as the possibilities to extend the half-life of the molecule. This allows less frequent hormone administration without compromising embryo yield (Gutiérrez-Reinoso et al., 2023). The outcomes of superovulation with a single long-acting rFSH injection comparable or superior to 8 twice daily injections of pFSH have been reported (Carvalho et al., 2014; Gutiérrez-Reinoso et al., 2022; Sanderson and Martinez, 2020), offering an improvement in the efficiency of superovulation protocols, and also, not to mention the improved animal welfare with the reduction of stress due to reduced handling.

eCG is frequently used in timed AI synchronization protocols and, to a lesser amount, in superstimulatory treatments. Compared to pFSH, the longer half-life of eCG allows only once or twice administration during superstimulation. Thus, in terms of half-life, recombinant eCG would not add value. However, the other benefits of producing eCG by the recombinant technology are, as with rFSH, higher purity and consistency of the product. The source of natural eCG is blood from pregnant mares during days 40-120 of pregnancy. Operations on these so called blood farms pose an animal welfare issue, which has raised concerns and criticism among the public (Manteca Vilanova et al., 2019).

As observed in recent years, the global embryo market is expanding, and the total number of embryos produced increased to 2,011,480 in 2022. Worldwide, IVP embryos accounted for 80.4% of all transferable cattle embryos in 2022 (Viana, 2023). Despite the evolution of OPU-IVP in recent years, these ARTs present some limiting factors that lead to variable results regarding blastocyst and pregnancy rates (Hansen, 2020), and thus directly influence commercial success. We will focus here in advances in techniques and selection methods to improve IVP and outcome.

### Culture systems

One of the main reasons for the poor results in IVP is the quality and origin of the oocytes. Aspirated immature oocytes are retrieved from ovarian follicles at different phases of the follicular growth and around 85 to 90% of these oocytes will reach the metaphase of the second meiotic division at the end of *in vitro* maturation. However, oocyte nuclear maturation is in many cases not accompanied by cytoplasmic maturation and full acquisition of developmental competence, which may result in fertilization and/or developmental deficiencies (Mermillod et al., 1999; Watson, 2007). Pre-maturation *in vivo* can be improved by advancing follicular growth with FSH administration followed by a period of coasting. Coasting, FSH withdrawal in the presence of endogenous LH, stimulates follicular differentiation and oocyte competence. These findings were confirmed by the higher developmental competence of oocytes derived from cows subjected to FSH treatment and coasting prior to OPU than those derived from untreated cows (Blondin et al., 2002; Luciano and Sirard, 2018). Several studies have suggested positive effects of exogenous LH injections and collection of *in vivo* matured oocytes on developmental competence, but more research is necessary to improve protocols (Egashira et al., 2019; Matoba et al., 2014; van de Leemput et al., 1999). Competence of oocytes *in vitro* may be enhanced by a period of pre-maturation in the presence of meiotic inhibitors (Chandra and Sharma, 2020). Meiotic arrest is induced by an increase in cAMP in oocytes, which is produced by the oocyte itself or supplied by the cumulus cells via gap junctions (Pan and Li, 2019). To improve the acquisition of developmental competence of oocytes, several researchers have cultured oocytes in conditions that prevent meiotic resumption by controlling cAMP concentration before IVM. For example, 3-isobutyl-1-methylxanthine (IBMX) is a non-specific phosphodiesterase inhibitor that prevents degradation of cAMP to 5’-AMP and inhibits meiotic resumption in bovine oocytes. Forskolin (FSK) is an activator of adenylate cyclase, which promotes the synthesis of cAMP (Thomas et al., 2002). Combining these meiotic inhibitors in pre-IVM has been shown to promote blastocyst rate and cell number in blastocysts (Albuz et al., 2010). Although other studies did not show a positive effect of oocyte pre-IVM (Guimarães et al., 2015; Mermillod et al., 2000), this methodology represents a promising direction for further research.

Currently, we place embryos on a polystyrene surface in a relatively large volume of medium and then leave them there in a static state. However, *in vivo*, the embryo is constantly moving within the female tract in a more viscous fluid and is exposed to a complex environment as it progresses from the oviduct to the uterus. There, the embryo is subjected to changes in oxygen and pH, and exchanges metabolites and signaling molecules with the female tract (Besenfelder et al., 2020). Future improvements to embryo culture systems will come not only from new improved media formulations but also from the shift from a static culture system to a more dynamic perfusion-based culture system. Microfluidics refers to devices and methods for the control and manipulation of fluid flows with length scales less than one millimeter (Stone et al., 2004). In the past decade, the study of microfluidics made it possible to create high-resolution devices for intracytoplasmic sperm injection, culture, and cryopreservation, paving the way not only for improvements in efficiency but also for automation of assisted reproductive technology (reviewed by Alias et al., 2021 and Sequeira et al., 2020). The implementation of microfluidics in *in vitro* culture has been shown to be beneficial for embryo development, as epigenetic reprogramming of these embryos is more similar to *in vivo* derived embryos (Ferraz et al., 2018). We will highlight two recent examples of the use of microfluidics in bovine IVP. Liquid Marbles (LM) is one type of 3D culture system, which is produced from highly hydrophobic particles that adhere to a drop of liquid, making a sphere that remain stable. The droplet size can be variable, allowing for group or individual cultures and requiring only very low amounts of culture medium (Aussillous and Quéré, 2001). A recent study investigated the effects of LM system on IVM and IVC independently. In the oocyte maturation experiment, no alterations in IVM rate were observed and the expression of transcripts associated with COC quality and development was altered in cumulus cells. In the embryo culture experiment, the blastocyst rate and number of blastocyst cells were lower than in the traditional IVP set-up and changes in global DNA methylation and hydroxymethylation were observed in resultant blastocysts. More research is needed to adjust this type of culture to improve blastocyst rates (Ferronato et al., 2023). Microdevices that utilize electric phenomena, such as electrowetting on dielectric (EWOD) technology, are recognized as a promising tool in ART (Karcz et al., 2022). This technology uses applied electric fields to manipulate microdroplets in a rapid manner on an array of insulated electrodes on a chip. The development and the first application of a microfluidic chip utlilizing EWOD in bovine IVP was recently presented (Karcz et al., 2023). Although challenges related to chip fabrication and application were encountered, the prototype sustained the cell cycles of embryos individually manipulated on the chips during IVC. In general, basic science and technological demonstrations have been developed, but more research is needed to commercialize microfluidics in the ART.

### Embryo selection

Improving laboratory culture conditions leads to increases in blastocyst and pregnancy rates. In addition, the ability to select the most viable embryo for transfer can reduce both time to pregnancy and pregnancy loss. Several invasive and non-invasive approaches for embryo quality assessment have been developed over the years to ensure the greatest pregnancy rate and finally the birth of healthy offspring. The methods used for the selection of oocytes and embryos were recently reviewed by Aguila et al. (2020), Rabel et al. (2023) and Wrenzycki (2021). In this section, we will discuss some of these recently developed techniques for embryo selection based on morphology.

Traditionally, embryos are graded based on morphological assessment according to the International Embryo Technology Society (IETS) standards for embryo evaluation (Bó and Mapletoft, 2018; Stringfellow, 2010). Although this method is simple and non-invasive, it is subjective and operator-dependent (Farin et al., 1995). It also gives little information on intracellular activity, such as metabolism, and it cannot quantify the percentage of fragmentation in the embryo, which may affect embryo viability (Thompson et al., 2016; van Soom et al., 2003). Therefore, new imaging techniques are needed to provide a rapid and objective assessment of embryo quality. Time-lapse imaging allows non-invasive selection of viable embryos based on morphokinetics, the pace at which stages of development occur, without removing them from an incubator for assessment (Gallego et al., 2019). A system has been developed to select bovine embryos for transfer using time-lapse monitoring. Several prognostic factors, such as timing of the first cleavage, blastomere number at the end of the first cleavage and blastomere number at the onset of the lag-phase, were identified to improve the prediction of viable embryos compared with conventional selection systems (Sugimura et al., 2012, 2017). However, time-lapse imaging is nowadays not integrated in commercial labs possibly due to practical and economic drawbacks. More research is needed to confirm that this technique may be a good predictor of the establishment of pregnancy. Although time-lapse imaging is used routinely in human IVF clinics, several studies concluded that morphokinetic data from human IVF embryos did not improve clinical pregnancy rates (Armstrong et al., 2019; Goodman et al., 2016). Additionally, imaging of bovine blastocysts can be challenging due to their dark cytoplasm (Jeong et al., 2009).

There are new ways, such as fluorescence lifetime imaging microscopy (FLIM) and hyperspectral microscopy, that are capable of assessing the quality of an embryo by using autofluorescence. Metabolic activity is related to embryo viability (Thompson et al., 2016), but quantitative assessment of the change in nutrients and metabolites (e.g. glucose, pyruvate, lactate and amino acids) requires specialized equipment and is time consuming. Interestingly, various endogenous biologic structures and molecules, many of which are involved in metabolism, are natural fluorophores which emit auto-fluorescence when cells are excited by light (Ramanujam, 2000). Examples of molecules that auto-fluoresce significantly are nicotinamide adenine dinucleotides (NAD(P)H) and flavin adenine dinucleotide (FAD). NAD(P)H and FAD activity can be quantified in oocytes and embryos using auto-fluorescence during fertilisation and embryo development (Sutton-McDowall et al., 2017, 2015). Based on these measurements, the REDOX state equation (FAD/(NAD(P)H + FAD)) of the cell can be calculated, indicating the level of metabolic activity of the embryo (Blacker and Duchen, 2016). NADH and NADPH have very similar excitation and emission wavelengths, making the separation of auto-fluorescence spectra between them difficult. FLIM can accurately separate fluorescence from these molecules based on the rate of quenching of their emission over time (Drozdowicz-Tomsia et al., 2014). Hyperspectral microscopy is a new application of autofluorescence where a large emission range is used to allow a broader spectral analysis of autofluorescence. This descriptive approach quantifies the differences in spectral patterning that are characteristic of some physiological change in cell behavior, such as apoptosis or cell differentiation, instead of identifying individual compounds (Gosnell et al., 2016). Sutton-McDowall et al. (2017) showed differences in the metabolic profile of morulas exposed to high or low O2 levels during culture using more detailed 18 channel hyperspectral microscopy. On the other hand, the two-channel autofluorescence technique was not sensitive enough to detect these differences. Hyperspectral microscopy is an important tool for discriminating embryo populations that are difficult to separate by morphology and less detailed metabolic measures alone. This approach may be an effective tool to combine with other diagnostic methods to provide an accurate assessment of embryo viability.

Until recently, live bovine embryos have not been evaluated based on their three-dimensional (3D) structure. Optical coherence tomography (OCT) has been developed in the 1990s for non-invasive 3D imaging and has been used commonly in ophthalmology (Wojtkowski et al., 2002). Advantages of OCT include its ability to image embryos without labels, and its use of low power light sources as compared to confocal systems, thus reducing the chance of damaging the embryo during observation. Caujolle et al. (2017) described for the first time 3D-imaging and intracellular movements of bovine blastocysts with OCT. They suggested that OCT could become a useful tool to quantify the level of damage sustained by cryopreserved embryos without the need for extended culture. More recently, Masuda et al. (2021) established a new protocol for live 3D-imaging of bovine preimplantation embryos and images were used to evaluate 22 parameters, such as the volume of the inner cell mass (ICM) and the thicknesses of the trophectoderm (TE). Although none of these parameters could be associated with successful pregnancy after ET, TE-related parameters may be useful for evaluating bovine embryos. Additionally, combining OCT measurements with time lapse imaging could make evaluation of other developmental landmarks, such as the timing of cleavage, timing of each developmental stage, and evaluation of cell numbers, more effective. This study also showed that OCT imaging did not affect embryo viability as conception and birth rates were similar as for regular ET. The same authors observed that the increase in the value related to the blastocoel along with the decrease in the value related to the thickness of TE and/or zona pellucida could be indicators for evaluating hatchability and, consequently, quality of IVP embryos (Masuda et al., 2023).

With the progress in the field of ’omics’, preimplantation embryos can be screened for entire genomes, transcriptomes, epigenomes, proteomes, and metabolomes. These tools can provide accurate information about thousands of potential biomarkers and the functional relationships between these biomarkers. As such, omics-related studies have been carried out extensively over the last three decades in search of the gold standard test for the evaluation of embryos before transfer or freezing and in an attempt to better understand the development of embryos prior to implantation (Chimote and Chimote, 2018; Jiang, 2023; Krisher et al., 2015). Many of these ’omics’ technologies have succeeded in discovering embryo quality markers. However, several of these “omics” are difficult to implement in field conditions. For example, genomic selection is nowadays performed on embryos by taking a biopsy, but only applied to a lesser extent as this may affect embryo viability and complicate the logistics. Compared to transcriptomics testing, proteomics and metabolomics are considered noninvasive as the culture medium is analyzed instead of the embryo itself. However, embryos must be individually cultured to analyze their individual secretomes, and the analysis requires expensive equipment and is time-consuming. Although most studies have described biomarkers associated with embryo quality, there is a lack of information on biomarkers associated with greater ability to establish pregnancy. A recent the study detected, using noninvasive methods, ions that had differential signal intensity, forming a profile, as well as two metabolites, pyruvate and lactate, that can be considered biochemical markers to identify IVP embryos with better pregnancy potential (Oliveira Fernandes et al., 2021). In conclusion, it is clear that while these tools are extremely useful for understanding pre-implantation embryo development, it is almost impossible for them to have direct in-field applications in embryo evaluation.

## Cryopreservation of semen and embryos

Preservation technologies are prerequisites for ART, with cryopreservation preferable when compatible with the gamete characteristics of the species. The cattle AI and breeding industry benefits from bovine cells, both embryos and spermatozoa, being relatively tolerant to cryopreservation protocols (slow, fast, vitrification) and storage in liquid nitrogen (-196 °C).

### Cryopreservation of semen

The plasma membrane of sperm cells differs from most other cell membranes in lipid composition with high amounts of polyunsaturated fatty acids (PUFAs) (Ladha, 1998; Mandal et al., 2014). Despite the fact that bovine semen tolerates freezing better than many other species, cryopreservation is in any case detrimental to the spermatozoa, leading to reduced fertilizing capacity. Freezing and thawing both contribute to reduced plasma membrane and acrosome integrity, reduced motility, and increased production of reactive oxygen species (ROS) (Yánez-Ortiz et al., 2022). Many studies have focused on the use of antioxidants in the freezing extenders to reduce the negative effects of ROS, reviewed by Amidi et al., focusing on different antioxidants’ effect on sperm cryoinjury and how antioxidants might improve sperm resistance to cryodamage (Amidi et al., 2016). Several studies have shown individual variability in sperm cryotolerance, and multidisciplinary approaches with sensitive omics technologies have been suggested for the discovery of biomarkers for semen quality and freezability (Hitit and Memili, 2022). As mentioned above, ML is a novel approach to reproduction research. Recently, ML was applied to predict liquid sperm quality in Holstein bulls, including e.g. climate conditions and the effect on sperm motility, morphology and sperm production (Hürland et al., 2023). Another approach to elucidate complex interactions between extender components was shown by applying statistical models for improved cryopreservation protocols (Mokhtassi-Bidgoli et al., 2023).

### Cryopreservation of embryos

Frozen-thawed embryos represented 44.3% of the cattle IVP transfers in 2022, maintaining the trend toward a greater use of cryopreservation of IVP embryos (Viana, 2023). A major challenge of assisted reproduction is the improvement of embryo production and cryopreservation protocols so that satisfactory rates of embryo survival and pregnancy establishment can be achieved after embryo cryopreservation. Currently, there are two methods of cryopreservation: slow-freezing and vitrification. Both techniques have advantages and disadvantages in terms of their application. Slow-freezing allows for direct transfer of embryos, but ice crystal formation remains an issue. This problem has been overcome by vitrification, but direct transfer is not possible with this method. Currently, the most popular cryopreservation method for IVP embryos is still vitrification, although slow freezing is increasing in popularity (Gómez et al., 2020). Despite the reduced competence of IVP embryos compared to *in vivo* derived embryos (Wrenzycki, 2018), there are nowadays satisfactory results for blastocyst rates, pregnancy rates, and less pregnancy loss (Demetrio et al., 2020).

To achieve successful application of vitrification in field conditions, the procedures used for the warming and transfer of the cryopreserved bovine embryo should be simplified. Due to the high concentrations of cryoprotective agents (CPA) required for vitrification, warming of vitrified embryos requires the removal of CPA in several dilution steps. This procedure requires laboratory equipment and trained personnel, which restricts the use of this technique on a large scale. Lately, efforts have been made to develop a vitrification–warming–transfer protocol that requires the same level of skill as for AI. Several devices have been used to warm and dilute vitrified embryos in a single step, varying from 0.25 ml plastic straws (Zhang et al., 2015), modified plastic straws (Ha et al., 2014), hand-pulled glass micropipettes (Vieira et al., 2007), Cryotops (Morató and Mogas, 2014), Cryotec (Tajimi et al., 2018) or Fibreplugs (Caamaño et al., 2015). Although these methods have shown acceptable hatching rates after warming of vitrified IVP embryos, little data is available on pregnancy rate after embryo transfer. So far, pregnancy rates for such vitrified embryos varies between 20 and 55% (Inaba et al., 2011; Vieira et al., 2007). Recently, an in-straw warming protocol was developed that is compatible with most vitrification devices for embryo transfer without sucrose gradient steps and embryo evaluation. The authors observed pregnancy rates that were similar between vitrified-warmed embryos and fresh embryos (40% vs 43%, resp.) (Oliveira et al., 2020). Although these results are promising, more data on embryo survival rates and subsequent pregnancy rates are needed to validate these new protocols.

Slow-freezing of embryos, despite having slightly higher operating costs, is more practical because it allows for direct transfer after thawing. In addition, lower CPA concentrations are required, reducing toxicity to the embryo (Voelkel and Hu, 1992). Moreover, efforts are made to improve slow-freezing protocols to achieve satisfactory pregnancy results. Sanches et al. (2016) showed that slow-freezing is also a good option for the cryopreservation of IVP bovine embryos. Embryos produced *in vitro* with sexed semen caused similar pregnancy rates on day 90 after their vitrification (31.2%) or slow-freezing (34.7%) and direct transfer (Sanches et al., 2016). This suggests that both cryopreservation techniques can yield similar pregnancy rates. Interestingly, a meta-analysis of more than 40 manuscripts was performed to elucidate the effects of the cryopreservation method on *in vitro* and *in vivo* survival of *Bos taurus* embryos. It was demonstrated that vitrification has a comparative advantage to temporally protect a proportion of embryos from cryodamage at early stages of embryonic development as compared to slow-freezing. However, pregnancy rates after embryo transfer are similar across the methods of cryopreservation (Arshad et al., 2021).

It is of the utmost relevance to the breeding industry that oocyte and embryo processing procedures are advanced in tandem with the progress in cryopreservation techniques. Enhancement of oocyte developmental competence and embryo culture, in combination with selection of good quality embryos, are essential in the improvement of cryopreservation and subsequent establishment of pregnancy. This has been extensively discussed in other reviews such as (Do et al., 2019; Ferré et al., 2020; Mogas, 2018; Valente et al., 2022).

Recently, a new class of CPA solutions was introduced based on graphene oxide nanoparticles. The low heat conductivity of the traditional CPA solutions surrounding embryos slows down cooling and heating rates of the embryo, imposing the risk of cryodamage. These new cryosolutions based on graphene oxide nanoparticles offer drastically increased thermal conductivity and lower viscosity (Hajjar et al., 2014). The improved thermal conductivity of these new CPA solutions resulted in similar re-expansion, hatching, and implantation rates of post-vitrification mouse embryos while also preventing an array of cellular and molecular stresses (Fayazi et al., 2022). No similar research has been performed on bovine embryos, but this could be a promising direction to improve the vitrification protocol. Utilization of gold nanorods (GNRs) is another recent technology that has been implemented in warming process after vitrification of zebrafish embryos. Khosla et al. (2017) micro-injected the CPA propylene glycol into zebrafish embryos along with GNRs and vitrified the embryos. They were able to warm the embryos rapidly (1.4 × 107 ºC/min) by irradiating the sample with a 1,064 nm laser pulse for 1 ms. This rapid warming process led to the outrunning of ice formation, which can damage embryos (Khosla et al., 2017). This nanoparticle-based warming process can potentially be used for bovine embryos.

Interestingly, the male can have an effect on the cryotolerance of the embryo. In a retrospective study of more than fifty thousand embryo transfers by a commercial IVP lab, a variation among different sires following the transfer of fresh and cryopreserved IVP embryos was observed. Pregnancy rates diverged between sires from 28.3 to 52.5% for fresh and 7.7-61.6% for cryopreserved embryos. Remarkably, some given bulls have very high pregnancy rates after transfer of cryopreserved embryos compared to fresh embryos and vice versa (Gonçalves et al., 2021). This intrinsic genetic effect in the expression of pregnancy rate shall make for interesting further investigations and could be a selection criterion to in- or exclude bulls in embryo production or determine if embryos should be transferred fresh or frozen.

## Artificial Intelligence in animal ART

Artificial intelligence (AI) is one of the most disruptive technologies at this point in time, and it also has great potential in ART. In the following, we will describe the technology and discuss the main challenges related to the application of artificial intelligence in ART. AI refers to the ability of a machine or computer to perform tasks commonly associated with intelligent beings. Machine Learning (ML) is a direction within artificial intelligence and refers to methods that can automatically learn from data. In this work we will use the term ML for both AI and ML. The two main parts of ML are supervised and unsupervised learning. Supervised learning refers to methods that learn from datasets where the answer, or label, is given for each observation. An observation could for example be data from IVC (e.g. a blastocyst image) and the label if the embryo resulted in pregnancy or not. Given data from another IVC, where the label is unknown, the trained ML model can be used to predict the label. Unsupervised learning, on the other hand, refers to methods that look for patterns in unlabeled data. One can, for example, imagine automatically grouping blastocyst images based on visual (morphological) characteristics.

Artificial Neural Networks (ANNs) represent an important group of supervised learning methods that are inspired by how our own brain works with neurons and synapses. Deep Neural Networks, or Deep Learning (DL), refer to especially large and complex ANNs and thus have more similarities to the complex human brain. A particularly attractive property of DL methods is that they can learn from unstructured data such as images or text without any human intervention. Research in fields consisting of unstructured data such as computer vision (images and videos), natural language processing (text) and speech recognition (sound and text) has yielded promising results, in many cases demonstrating that DL can to outperform other ML methods, and perform as good as or even better than humans in certain scenarios.

ML-based methods have recently become very popular in medicine, mainly due to deep learning. ML is for example used in cardiology to detect atrial fibrillation (Itchhaporia, 2022), in gastroenterology to process images from endoscopy do detect abnormal structures (Le Berre et al., 2020) or in neurology to detect clinical stroke (Leslie-Mazwi and Lev, 2020). Within the field of ART, articles trying to give an overview and explain the basics of ML are also emerging (Ratna et al., 2020; Rosenwaks, 2020).

General for ML in ART Fernandez et al. (2020) provide an overview of different ML algorithms and their potential uses in human IVF clinics, specifically focusing on the classification of embryonic cells and semen samples. Their review indicates that most algorithms have achieved satisfactory precision, demonstrating the potential of a wide range of ML techniques in the field of ART. Moreover, Raimundo and Cabrita (2021) described the potential of an ML system to predict IVF outcomes, covering the entire workflow of IVF treatments. They emphasize the need for such systems to decrease interobserver variability and improve medical productivity.

More specifically, in the area of ART for cattle, the study by Bremer et al. (2023b) demonstrates the potential of automating SC measurements in bulls using 3D imaging and convolutional neural networks, as described in the first part of this paper. Among the studies exploring precision livestock farming (PLF) applications, Curti et al. (2023) present a comprehensive overview of the ways sensors, computer vision (CV), and machine learning (ML) algorithms are being integrated into animal production and reproduction systems. Their work underscores the transformative potential of these technologies across various domains, including estrus detection, sperm and embryo evaluation, feed management, health monitoring, and overall production optimization. Importantly, the authors delve into the practical challenges faced throughout the PLF pipeline. They emphasize that data quality, often influenced by collection methods and animal variability, is a critical determinant of ML model performance. Curti et al. (2023) further note that connectivity constraints in remote production systems, along with the need for adaptable data storage solutions, can hinder the seamless implementation of PLF applications. They advocate for careful attention to data preprocessing, feature extraction, and model selection alongside the development of intuitive user interfaces to ultimately drive the meaningful adoption of PLF solutions.

In the areas of optimizing livestock AI, Zuidema et al. (2021) delve into the transformative potential of advancements in semen analysis, sperm selection, and storage techniques. They address the diverse ways in which AI is employed across livestock sectors, emphasizing how species-specific challenges and production goals influence choices such as frozen vs. liquid semen usage. A core area of improvement emphasized by the authors is the transition towards objective semen analysis using technologies such as CASA and, notably, biomarker-based flow cytometry (FC). These methods go beyond traditional morphology and motility assessments, providing insight into sperm DNA integrity, acrosomal status, and other parameters directly related to fertilization potential. Zuidema et al. (2021) further underscore the promise of sperm selection protocols such as colloid centrifugation and nanopurification. By using biomarkers associated with sperm dysfunction, these tools enable improved sperm quality with the potential to increase AI success even when utilizing semen from males of variable fertility. While addressing broader AI trends, the authors also acknowledge the ongoing development of sperm sexing for non-cattle species, which offers unprecedented control over offspring sex ratios. Finally, they discuss the continued pursuit of more efficient semen extenders and cryopreservation methods to address challenges for species where frozen semen protocols are less successful.

### Main challenges for efficient ML

The current state of research in the field of ML applied to ART in cattle shows a diverse range of advancements and challenges. The main obstacles that we need to overcome at this stage are:

- Proper evaluation and testing of ML systems in relation to outcomes and regulations;
- Large and open datasets that can be used to reproduce results and train basic ML models that can be fine tuned for specific applications;
- A better understanding of the technical aspects and how to determine the performance of ML systems so that it is possible to assess their practical value in the field/lab. For this we need to shift away from proof of concept studies to large trials;
- How can the use of ML in ART be standardized to enable more transparent, comparable, and reproducible lab results?

#### Data availability, comparison, evaluation and why interdisciplinary collaboration is the key

In general, evaluating an ML-based system requires us to have a dataset that is split into a training, validation, and testing part. The training partition would be used to train the algorithm, the validation to evaluate if the algorithm works on unseen data, and the testing part would be used at the very end to make the final evaluation of the system. Ideally, the data set should contain data from different labs. The testing data should be from a different site than the training and validation data to ensure that the algorithm can generalise to data collected from different locations. In practice, most current work does not have access to enough data and often relies on using validation data for evaluation without an independent test dataset (Butola et al., 2020; Fukunaga et al., 2020; Mirsky et al., 2017; Zhao et al., 2021). To compensate for the lack of test data, most studies use cross-validation (training and testing is performed on different splits of the dataset and then averaged). This is not optimal and will not measure the clinical performance of the tested model but rather the performance of the algorithm on the specific data at hand. This can still help determine the differences between different algorithms but not for generalisability and robustness (which are both important for clinical practice). One does not want to end up with an algorithm that cannot handle data that one did not see before.

In addition to the data and technology-related challenges, the design of the evaluation is also important. We must evaluate the methods using metrics and measurements that are appropriate for the task at hand. For example, do we want to determine embryo quality based on each frame extracted from the video, or do we want to do it per embryo. The first one would lead to better results since there are many near duplicates in a video that would make it easy for the ML system to achieve high scores, whereas the latter one is more clinically relevant, harder, and would require more videos for training and evaluation.

All the challenges from dataset design (bias, splitting, size, quality) to model evaluation (testing method, metrics used) show a need for standardization. Besides, this also indicates that close collaboration between practitioners and technical persons is required. We are at a point when these two have to work closely together from the very start (before data is collected) to the very end (evaluation via clinical trial). At all stages of developing a complete ML system, both sides have important input to give. The data scientists can help shape real and relevant medical research questions, support planning and data collection. The practitioners can provide input to the model design by giving insight into how they approach corresponding scenarios. Current ML systems allow us to design an algorithm that can mimic the way humans would solve a specific task. To do that, one needs to have a good understanding of these processes. Here is the data, and see what you can do with it is not enough. The whole ART community needs to get involved in the design and testing phase at an early stage and work on a common standard. We also make a distinction between basic and field studies. Both have their relevance and usefulness. Preclinical studies are essential to compare different state-of-the-art methods and push the field forward, whereas large field studies are important to determine the quality and clinical usefulness of certain methods. Both need to be used and interact interchangeably.

#### Transparency, explainability and ethics

Most current ML systems, especially the ones based on deep learning, are black boxes. This means that they make their decisions based on millions of parameters without the possibility to explain why a prediction was made. Making decisions without the ability to justify them is unacceptable to the breeders. There is a lot of research going on in increasing the explainability of these black boxes (Barredo Arrieta et al., 2020; Holzinger et al., 2019). This research also slowly arrives in the medical ML applications and AI in ART focusing mainly on very simple visualization methods (Abbasi et al., 2021; Liu et al., 2020). But only explaining the decision of an ML system is not enough to build a genuinely transparent system (Weller, 2019). The whole pipeline of these systems needs to be transparent and open. This includes describing how the data was collected, what is included in the data, how the system was trained, how the system was evaluated, and how the explanations were produced. Highly accurate ML systems trained on very little data from one laboratory will probably not generalise well to unseen data (Sun et al., 2017). Data transparency and openness are essential parts of understanding the real value of ML, and more data transparency is crucial for the success of ML in ART. In addition, the clear effect on clinical decisions such as choosing spermatozoa for ICSI or choosing the right embryo for transfer can only be fully understood when we know all details of the ML system in addition to clinical trials.

We can also observe the emergence of commercial ML systems in high-impact journals with almost no details about any parts of the system (Agarwal et al., 2019), mainly focusing on positive results. This can lead to producing many black-box systems making claims without any clear scientific evidence or possibility to reproduce results or findings. Even less well- performing ML systems can be presented in a way that they appear superior to others. This can be influenced heavily by the selection of metrics, data, and evaluation methods. Even well-established metrics that are commonly used in medical studies, such as specificity, ROC, and sensitivity, can be used in ways that can obfuscate results. Thus it is also important to be transparent on the choice, presentation, and interpretation of metrics (Saito and Rehmsmeier, 2015). If studies provide as much transparent information as possible about the metrics, such as report confusion matrices in addition to the calculated metrics. In that case, others can easily verify the reported metrics and calculate others that may not have been reported.

To assess the clinical performance of ML systems, the community needs to agree on a common minimum standard of how ML systems should be described. At least the design and development process, the data (training, validation, and testing), the testing scenarios, and the evaluation metrics should be described in detail with a focus on clinical relevance (like in Bori et al., 2020; Javadi and Mirroshandel, 2019). For preclinical studies that focus on comparing different ML methods or introducing novel methods, the sharing of the model, source code, and data should be highly encouraged. This is important to ensure comparable and reproducible science. Furthermore, researchers should consider including explainable ML methods by using them to understand and discuss specific cases where a model failed. Failure analysis will add another layer of evaluation to avoid publishing studies with obvious errors and show the limitations of ML systems (Zech et al., 2018). More transparency in the entire ML process in ART will allow a better and more efficient discussion of legal aspects, making these systems usable in practice.

#### Standardization and benchmarking of ML in ART

The standardization of ML in ART needs to include specific requirements for it to be truly valuable. First of all, there is the need for a common standard for evaluating performance and transparency. This will be difficult to standardize but requires the involvement of the communities and journals in ART, in addition to an evaluation through field trials. Furthermore, we need a common way of benchmarking and comparing different systems. In computer science, this is often done using open benchmarking datasets collected and curated by the scientific community. This allows for better comparability and reproducibility, and is essential to enable continuous retesting of ML systems. ML systems are dependent on data that is collected from different pieces of hardware. If the hardware changes, like data collected at higher resolutions, the systems will have to be evaluated on the data collected from these new devices. It is not given that a model will work the same on images with different resolutions or videos with different frame rates. This means we need these community-wide benchmarking datasets to continuously test before, during, and after clinical trials to verify the performance.

The datasets themselves also need to be continuously updated to follow the technological advances and new medical findings. There exists a few open datasets in ART for animals, even less for specific animal groups such as cattle.

Available datasets from studies in cattle:

- Extenders (egg yolk, soya bean lecithin and liposome) for cryopreservation of dairy bull semen (Miguel-Jimenez et al., 2020);
- The association between subclinical mastitis around calving and reproductive performance in grazing dairy cows (Villa-Arcila et al., 2017).

Open datasets are a good start, but they need to be larger, more diverse, and continuously updated over time. This would make them similar to a rolling gold standard. In the optimal case, we would have one publicly available dataset that can be used for developing algorithms and a hidden test dataset that can be tested on. This would ensure that we have a common standard for training and testing, which allows reproducible and comparable results that are necessary to make ML in ART clinical relevant and valuable. It would also allow the whole community to better determine and separate high-quality from low-quality studies and enforce a certain standard on the industry, using the same bar to verify their products.

## Conclusion

The cattle breeding industry benefits from an extensive arsenal of technologies within ART, ranging from time-honoured techniques to the latest advances. In particular, omics technologies are establishing their value as they provide vast amounts of data on the impact of ART on gametes. New tools are being developed to select and process gametes more efficiently, but more research is needed to implement these tools in field or laboratory conditions and to confirm their superiority compared to current techniques. Practitioners and engineers should collaborate more closely as technologies as 3D-imaging and microdevices are promising tools for objective selection of gametes and automation of culture systems. Currently, ML is leading the charge in enhancing precision and efficiency within ART. Beyond simply improving the accuracy of the evaluation of semen and embryos, the aim is to improve the fertility results, which will ultimately manifest itself in the successful birth of healthy and live offspring.

Looking ahead, the potential of ML to transform the industry is profound. By aggregating and analysing pregnancy data from diverse sources and studies, ML holds the promise of conducting “real-time meta-analyses.” Such a capability could uncover hidden associations and correlations within the data, a task made challenging by the binary nature of pregnancy outcomes and the myriad of variables and confounders at the farm level. Traditional hurdles to achieve statistical significance in insemination or embryo transfer studies due to these complexities could be overcome with the power of ML. Thus, ML not only aims to enhance decision-making but also aspires to ensure a more efficient use of resources, reducing the reliance on extensive biological materials. This foresight into the role of ML underscores a transformative shift toward more informed, data-driven practices in cattle breeding, promising significant advances in fertility research and applications with fewer resources.
